# Potential overestimation of HIV-1 sub-subtype F1 circulation in Rio de Janeiro, Brazil

**DOI:** 10.1590/0074-02760170483

**Published:** 2018-06-11

**Authors:** Bianca Cristina Leires Marques, Mariza Gonçalves Morgado, Monick Lindenmeyer Guimarães

**Affiliations:** Fundação Oswaldo Cruz-Fiocruz, Instituto Oswaldo Cruz, Laboratório de Aids e Imunologia Molecular, Rio de Janeiro, RJ, Brasil

**Keywords:** HIV-1, sub-subtype F1, full-length genome, molecular epidemiology, genotyping

## Abstract

In Brazil, detection of the HIV-1 sub-subtype F1 has decreased with a simultaneous increase in detection of the recombinant FB and FC forms. In previous HIV-1 *env* molecular epidemiology studies in Rio de Janeiro, 11.4% of the detected sequences were of the F1 sub-subtype. With the goal of re-estimating the prevalence of the HIV-1 F1 sub-subtype, we performed extended analyses of these samples by examining five genomic regions, resulting in 3.3% being confirmed as F1. Moreover, genomic analysis of 11 of the 21 samples identified as F1 confirmed that nine were F1 and two were BF1. Considering the number of samples assayed, the prevalence of F1 was quite low, which supports the use of different genomic regions for the assessment of HIV-1 classification in countries where several subtypes and recombinant forms co-circulate.

The HIV-1 group M is responsible for approximately 90% of HIV infections worldwide (Hemelaar et al. 2011). Based on its genetic variation, this group can be divided into nine subtypes (A-D, F-H, J, and K) that exhibit 17-35% and 8-17% of intra- and inter-subtype variation, respectively, depending on the genomic region analysed. The HIV-1 subtype F was initially described by Dumitrescu in 1994 (Dumitrescu et al. 1994) using samples from Romanian HIV-1 infected individuals and simultaneously from Brazilian individuals (Morgado et al. 1994). Posterior analysis of the African sequences allowed for the reclassification of this subtype into sub-subtypes F1 and F2. The F1 sub-subtype has been identified in European (Romania, Italy, Spain and Russia) and South American countries (Argentina, Chile, Bolivia and Brazil), while F2 is mostly restricted to Central African countries (Hemelaar et al. 2011).

Based on compiled data from 2000 to 2007, the prevalence of HIV-1 sub-subtype F1 was estimated as 0.45% worldwide (Hemelaar et al. 2011). Among the 6948 complete HIV-1 genomic sequences present in the Los Alamos HIV database (http://www.hiv.lanl.gov/, accessed in August, 2017), only 58 (0.8%) belong to the F1 sub-subtype, 15 (0.2%) of which are from Brazil. In addition, this sub-subtype is reported in 15 of the 90 (17%) HIV-1 circulating recombinant forms (CRFs) already described, frequently in association with the B subtype. Of these 15 CRF_BF1, nine were described in Brazilian individuals, and only one was reported outside of South America (http://www.hiv.lanl.gov/content/sequence/HIV/CRFs/CRFs.html, accessed in August, 2017).

Previous molecular epidemiological studies from Rio de Janeiro attempted to describe the prevalence of HIV-1 sub-subtype F1 and BF1 recombinants based on only small genomic segments, typically from the *env* (C2V3) or *pol* (PR/RT) genes. Regarding the C2V3 segment, estimates of the prevalence of sub-subtype F1 ranged from 9.3% to 24.4%, while the prevalence of BF1 recombinants was estimated to be approximately 1%. For the PR/RT region, the prevalence of sub-subtype F1 varied from 4.9 to 22.2%, while that of the BF1 recombinants ranged from 3.3% to 14.8%. Although the first description of the HIV-1 BF1 recombinant strain was based on the analysis of the *env* gp120 V1-V5 fragment (Sabino et al. 1994), further descriptions were typically based only on the viral *pol* gene. Studies based on the *env* gene detected a higher prevalence of sub-subtype F1 than of the BF1 recombinants, and the opposite could be verified in studies based on the PR/RT region. Thus, to obtain a more accurate estimate of the frequency of sub-subtype F1 in the Rio de Janeiro HIV-1 epidemic, we characterised five genomic regions of sequences obtained from HIV-1 F1 samples, which were previously identified based only on sequence analysis of the C2V3 region of gp120. In addition, the complete genomes from some of the samples were sequenced.

Seventy-three HIV-1 sub-subtype F1 isolates were identified in previous HIV-1 molecular epidemiological studies carried out at the Laboratory of AIDS and Molecular Immunology of Oswaldo Cruz Institute/FIOCRUZ. Included in these previous studies were 635 individuals from Rio de Janeiro, Brazil, from whom biological samples were collected between 1998 and 2013 and were screened using the C2V3 envelope region (Teixeira et al. 2004, Guimarães et al. 2010, Pimentel et al. 2013, Guimarães et al. 2015, Leite et al. 2017). From those 73 samples, biological material was still available for only 55 individuals; these samples were used in the present study. This study was approved by the Evandro Chagas Nacional Institute of Infectious Diseases of Oswaldo Cruz Foundation (INI-FIOCRUZ) Ethical Research Committee (Ethics Committee CAE: 03925112.0.000.5248) as an anonymous unlinked study.

The extracted DNA was assayed by nested polymerase chain reaction (PCR) and sequenced, covering five regions of the HIV-1 genome: *gag* (nt 836-1844 relative position to HXB2), *pr/rt* (nt 2253- 3555 relative position to HXB2), *int* (nt 4173- 5213 relative position to HXB2), *env* (nt gp41/gp120- 6817-8277 relative position to HXB2) and *nef* (nt 8698-9474 relative position to HXB2). The chromatograms were edited and assembled using SeqMan from the DNASTAR package. The sequences obtained for each genomic region were assayed using Clustal W in MEGA 6.0, and the phylogenetic analyses were performed using the neighbor-joining (NJ) method and the Tamura-Nei substitution model. Reference sequences for the HIV-1 group M subtypes (A-D, F, H, J and K) were obtained from the Los Alamos HIV database (http://hiv.lanl.gov/). Bootstrapping (1000 re-samples) was used to evaluate the reliability of the phylogenetic tree topology. All samples were assessed by recombination analysis using the bootscanning method as implemented in Simplot 3.5.1, with the following parameters: 200-nt window, 20-nt increments, NJ method with Kimura's two-parameter correction, and 100 bootstrap replicates.

A subset of samples classified as sub-subtype F1 for all HIV-1 genomic regions analysed were further assayed by full-length amplification using four overlapping fragments amplified by nested PCR, as previously described in Reis et al. (2017).

Genomic sequences were determined using 1 ng of an equimolar mixture of the four amplified fragments. They were subsequently sequenced using the Illumina HiSeq2500 platform following the manufacturer's protocol (Illumina^®^, San Diego, California, USA). The raw data were processed using Trimmomatic to exclude adaptors. Only bases with Q>30 in Sickle were considered for assembling the genomes. After genome assembly, the consensus genomic sequences obtained were subjected to phylogenetic and recombination analyses, as described above.

From the fifty-five HIV-1 sub-subtype F1 samples analysed in this study, all five genomic fragments (*gag, pol* (PR/RT), *int, env* (gp120/gp41) and *nef*) were amplified from thirty-four samples (71%), while four (n = 15) or three fragments (n = 6) were amplified from the remaining samples.

Phylogenetic trees were reconstructed for each of the five genomic regions (Fig. 1), and each fragment was subtyped based on phylogenetic and recombination analyses. Although some (< 80) were detected in clade branches, removal of the recombinant samples allowed for an increase in these values to > 80 as follows: GAG (F1 from 38 to 97 and B from 58 to 99), PR/RT (F1 from 12 to 82 and B from 42 to 83), INT (F1 from 79 to 90 and B from 28 to 81), ENV (F1 from 55 to 99 and B 95 to 98) and NEF (F1 from 37 to 99 and B from 8 to 80). Recombinant sequences were detected in *gag* (36%), *nef* (33%), *pol* (PR/RT) (28%) and *int* (22%). The final subtype classification of each sample was a result of the analysis of the crossing data of all studied genomic regions. Twenty-one out of 55 samples could be classified as “pure” HIV-1 sub-subtype F1, and 34 could be classified as recombinants (32 BF1, one BUF1 and one CF1). To determine if some of the sequences could be assigned as a CRF_BF1, as already described, we performed a phylogenetic analysis of the PR/RT fragment. This analysis included all CRF_BF1 classified as F1 in the C2V3 region, since this was the selection criterium for the samples to be included in this work (Fig. 2). Although three sequences clustered together with a given CRF (1 CRF12_BF, 2 CRF39_BF), they did not have the same breakpoints along the genomic fragments analysed (Fig. 2), indicating that they were unique recombinant forms (URF). Of the 55 samples assayed, 20 were collected from 1998 to 2002, nine (45%) of which were characterized as HIV-1 sub-subtype F1 and 11 (55%) of which were characterised as BF1 recombinants. The remaining 35 samples were collected between 2008 and 2013, of which 12 (34.2%) were classified as the HIV-1 F1 sub-subtype, 21 (60%) as BF1, one (2.9%) as CF1 and one (2.9%) as BF1U. The decrease in HIV-1 sub-subtype F1 and the increase in recombinant forms between these two time intervals were not significant.

**Fig. 1:**
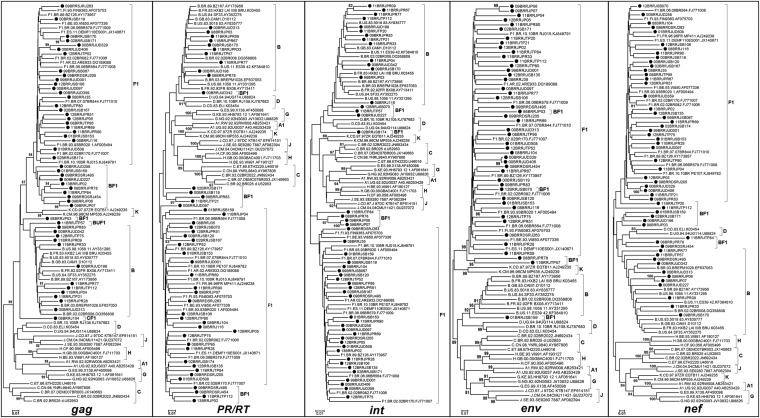
neighbor-joining phylogenetic analysis of the 55 studied HIV-1 samples in five genomic regions: *gag*, 854 bp; *pol* (PR/RT), 949 bp; *int*, 672 bp; *env*, 1416 bp and *nef*, 459 bp. The query sequences are indicated by black circles. Only bootstraps up to 80% were considered. Clades were defined based on data from phylogenetic and bootscan analyses.

**Fig. 2:**
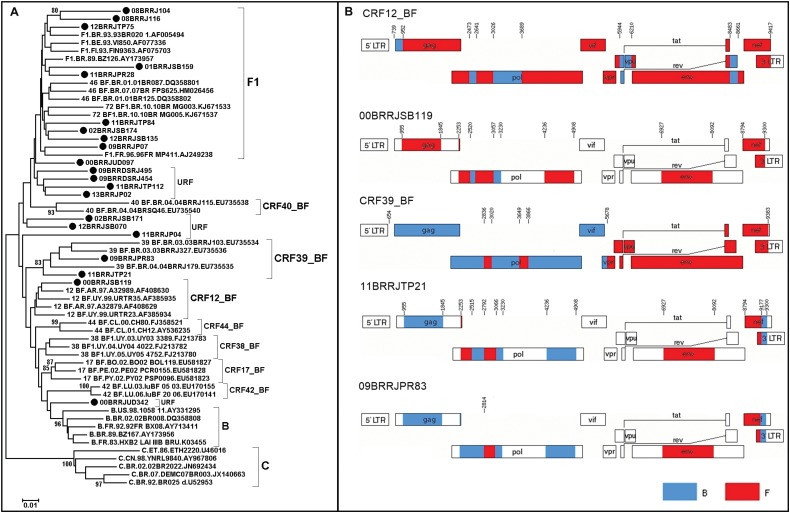
(A) neighbor-joining phylogenetic analysis of the PR/RT region from all BF1 and F1 samples studied in *pol* and all CRF_BF that were the F1 sub-subtype for the C2V3 region. The query sequences are indicated by black circles. Only bootstrap values higher than 80% were considered. (B) Schematic representation of the CRF12_BF and CRF_39 breakpoints, including those from the three samples that clustered together, are depicted.

After characterising these samples for least three HIV-1 genomic regions and with the goal of making a more accurate estimate of the presence of HIV-1 sub-subtype F1 in our samples, we used the following rationale. Originally, 635 samples were characterized based on the C2V3 *env* region, of which 74 (11.6%) were classified as the F1 sub-subtype but only 55 (75%) had biological material available for further testing. The reanalysis of these 55 samples using at least three genomic regions revealed that only 21 samples (38%) were confirmed to be F1. These results led to an overall estimation of 3.3% (21/635) to 4.3% (≈ 28/635) for this sub-subtype, which was calculated based on 38% of the original 74 (n = 28) C2-V3 sub-subtype F1 samples and on the sub-subtype F1 prevalence observed in our sub-sampling. The HIV-1 F1 frequency estimation based on each of the studied regions was as follows: *gag* (3.9%), *pr/rt* (4.2%), *int* (4.6%), and *env* (6.5%). Alone, none of the five regions analysed permitted an accurate estimation of the frequency of the HIV-1 sub-subtype F1.

The 21 samples characterised as containing HIV-1 F1 in at least three genomic studied regions of the genome were further assayed by full genome amplification, by using next generation sequencing (NGS). Of these, 11 (52.4%) were successfully amplified and sequenced. Nine of the 21 samples were confirmed as containing pure HIV-1 sub-subtype F1, and two contained URF_BF1 (Fig. 3), leading to a reduction in at least 18% (2 out of 11) of our estimates.

**Fig. 3:**
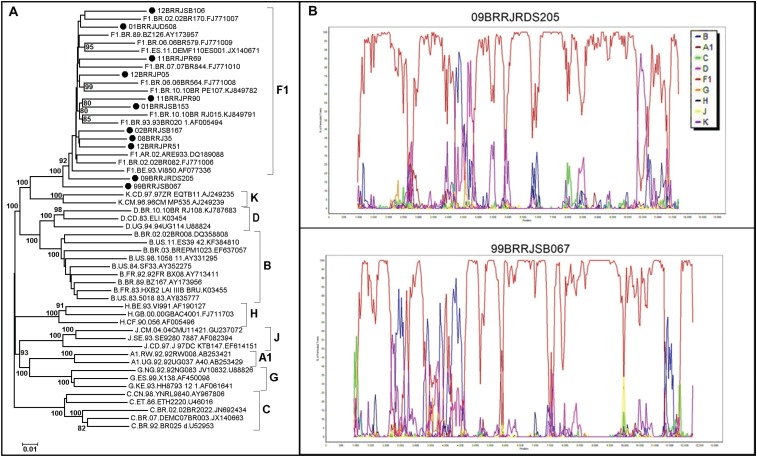
(A) neighbor-joining phylogenetic analysis of the 11 HIV-1 full-length genomes. The query sequences are indicated by black circles. Only bootstrap values higher than 80% were considered. (B) Bootscan analysis (200 nt window, 20 nt increments, NJ method with Kimura's two-parameter correction and 100 bootstrap replicates) of the recombinant samples 09BRRJRDS205 and 99BRRJSB067.

Considering the results of the classification based on the five genomic regions and on the full-length genomes, we could estimate that the frequency of HIV-1 sub-subtype F1 in our samples is approximately 3%.

Most HIV-1 molecular epidemiological studies are based on the characterisation of only one genomic region, which does not allow an accurate estimation of the prevalence of the HIV-1 subtypes and recombinant forms, resulting in overestimation or underestimation, primarily in countries where distinct subtypes co-circulate (Thomson and Nájera 2005, Hemelaar et al. 2011). Only full-length HIV-1 genomic analysis can determine the most likely HIV-1 diversity in a population, as proposed by Hemelaar et al. (2011). Based on the compiled molecular epidemiological data from partial or full genomic sequences from 2004 to 2007, HIV-1 recombinant sequences (URFs and CRFs) accounted for 20% of HIV-1 forms (Hemelaar et al. 2011), and until now, 90 CRFs have been described in the Los Alamos database (http://www.hiv.lanl.gov/content/sequence/HIV/CRFs/CRFs.html, accessed in August 2017). Taken together, this information reflects the increased importance of the recombinant forms in the HIV-1 pandemic. Thus, changes in HIV-1 diversity surveillance analysis are necessary (Hemelaar et al. 2011). However, obtaining full-length genome sequences is still expensive and laborious, even when applying NGS methods. To address this problem, in the present study, we investigated the use of five HIV-1 genomic regions to assess HIV-1 diversity, including highly recombinogenic regions [*pol* (PR/RT), *int* and *env* (gp41)] (Magiorkinis et al. 2003).

Previous molecular epidemiological studies revealed distinct HIV-1 subtype prevalence according to the analysed genomic region and depending on collection date and risk groups. The use of more than one gene or the full-length genome has allowed a smaller F1 prevalence (0.5 to 4.5%) to be observed (Guimarães et al. 2002, Sanabani et al. 2009, Sanabani et al. 2011). Based on our dataset from Rio de Janeiro, the frequency of HIV-1 sub-subtype F1 also varied according to the genomic region, ranging from 11.6% when using C2V3 *env* to 3.9% when using *gag.* Thus, when using *gag,* we would be able to identify 36% of the recombinants, while when using *pol* (PR/RT), the most used region due to it being an HIV antiretroviral target, we would be able to identify 28% of the recombinants. The use of *gag* and *pol* together allowed for a recombinant detection of 67%; however, the highest recombinant detection (72%) was obtained using *gag* and *nef*.

In conclusion, subtype classification based on more than three genomic regions, combined with the results based on full-length genomes, allowed us to estimate the occurrence of sub-subtype F1 of approximately 3% in our samples from Rio de Janeiro. This indicates an overestimation of the occurrence of this sub-subtype in previous studies based only on the C2V3 region sequence.

*Sequence data* - All nucleotide sequences are available from the GenBank database, with accession numbers as follows: ENV (MG365686-MG365721), GAG (MG365722-MG365760), full-length genomes (MG365761-MG365771), INT (MG365772-MG365808), NEF(MG365809-MG365851), and POL (MG365852-MG365882).
